# Musculature of the male genitalia in *Rivellia* (Diptera: Platystomatidae)

**DOI:** 10.3897/zookeys.545.6702

**Published:** 2015-12-14

**Authors:** Tatiana V. Galinskaya, Olga G. Ovtshinnikova

**Affiliations:** 1 Department of Entomology, Faculty of Biology, Lomonosov Moscow State University, Leninskie Gory 1–12, Moscow, 119234, Russia; 2 Zoological Institute, Russian Academy of Sciences, St. Petersburg, 199034, Russia

**Keywords:** Tephritoidea, Platystomatidae, male genitalia, sclerites, musculature, body size

## Abstract

The musculature of male genitalia was studied hitherto only in two species of Tephritidae, one species of Platystomatidae, one species of Pallopteridae, and three species of Ulidiidae of the superfamily Tephritoidea. The split of the hypandrium from one structure into three (the hypandrium and two lateral sclerites) is traced. The hypandrial origin of the lateral sclerites of the hypandrial complex is shown based on the localization of muscle attachment sites. The subepandrial origin of the inner lobes of the surstyli is also confirmed.

## Introduction

The signal flies (Tephritoidea: Platystomatidae) include nearly 1300 described species of more than 120 genera occurring predominantly in the Paleotropics, with a few genera and species in the Holarctic and Neotropic regions (V.A.Korneyev, unpublished data). They are a sister group to the Pyrgotidae + Tephritidae lineage, sharing with them numerous synapomorphies, including the structures of male genitalia, such as the phallus stored in rest in a membranous pocket under the 5^th^ tergite of abdomen, glans clearly separated from the remaining part of distiphallus, lateral (outer) surstyli fused to epandrium, phallapodeme and adjusting structures of hypandrium forming a “fultella”, etc. (Korneyev 1999).

The male postabdomen in Platystomatidae consists of modified segments 6–8 (pregenutal segments) and derivatives of segments 9–11 forming the genitalia and cerci.

In the superfamily Tephritoidea, the musculature of the male genitalia has been studied in representatives, shown in Table 1.

**Table 1.** Representatives of the superfamily Tephritoidea, in which the musculature of the male genitalia has been studied.

Species	Family	Reference
*Ceratitis capitata* (Wiedemann, 1824)	Tephritidae	Hanna (1938), Valdez-Carrasco and Prado-Beltran (1990)
*Campiglossa hirayamae* (Matsumura, 1916)	Tephritidae	Sueyoshi (2006)
*Rivellia basilaris* (Wiedemann, 1830)	Platystomatidae	Sueyoshi (2006)
*Temnosira trichaeta* Ozerov, 1993	Pallopteridae	Sueyoshi (2006)
*Timia erythrocephala* Wiedemann, 1824	Ulidiidae	Galinskaya and Ovtshinnikova (2015)
*Ulidia ruficeps* Becker, 1913	Ulidiidae	Galinskaya and Ovtshinnikova (2015)
*Physiphora alceae* (Preyssler, 1791)	Ulidiidae	Galinskaya and Ovtshinnikova (2015)

There are different views on the homology of some genital sclerites in the superfamily Tephritoidea. Different terminology is therefore used for some structures in a few cases, where morphology of Platystomatidae genitalia is considered: outer and inner surstyli (McAlpine 1973, 1999) or **lateral and medial surstyli** (White et al. 1999, Korneyev 2001); **lateral sclerites of hypandrium** (McAlpine 1973, White et al. 1999), gonites and vanes of fultella (Korneyev 1987), gonocoxites and vanes of phallapodeme (Korneyev 1999); decasternum or 10^th^ sternite (Korneyev 1987), ventral plate of proctiger, derivate of 10^th^ sternum (Ovtshinnikova 1989, 1994, 2000), **subepandrial sclerite** (Cumming et al. 1995) etc. Here, we provisionally follow terminology of White et al. (1999), with some reservations.

Study of the musculature is helpful not only in specifying the functions of genital sclerites, but also for revealing homology of some poorly traced structures (Ovtshinnikova 1989, 1993, Ovtshinnikova and Yeates 1998; Galinskaya and Ovtshinnikova 2015).

Recently, the musculature of the male genitalia was described for three species of the tribe Ulidiini (Galinskaya and Ovtshinnikova 2015) of the family Ulidiidae, which is a basal group to Platystomatidae + Pyrgotidae + Tephritidae lineage (Korneyev 1999).

In this paper, the musculature of male genitalia is described in *Rivellia* (Platystomatidae), continuing comparative study of morphology of the Tephritoidea.

## Methods

The terminology of the genital sclerites mainly follows White et al. (1999), Kameneva (2000), Galinskaya (2012), and Sinclair (2000).

Musculature of the male genitalia was studied by manually dissecting material (preserved fresh in 70% alcohol) with microknives in water under a Leica MZ9^5^ stereomicroscope. The illustrations were obtained using the image capture function of the Leica MZ9^5^ trinocular head and subsequently processed.

The male genital muscles of Tephritoidea were classified into several groups: muscles of the epandrial complex, muscles of the hypandrial complex, tergosternal muscles, and pregenital muscles. The muscles are designated by numbers following the classification previously accepted by Ovtshinnikova (1989).

List of abbreviations: cerc – cerci; epand – epandrium; bph – basiphallus; hypd – hypandrium; l scl – lateral sclerite of hypandrium; l sur – lateral surstylus; m sur – median surstylus; phapod – phallapodeme; sbepand scl – subepandrial sclerite; sec scl – secondary sclerotisation of hypandrial membrane; sur – surstylus; 8 stgst – 8 syntergosternite; M1–M19 – muscles.

## Results
Platystomatidae

### General plan of male genitalia

Male genitalia are similar to those in Tephritidae: hypandrium U-shaped, with membranous fold posterior of basiphallus, but without epiphallus, metaphallic plate, or a sclerotized bridge connecting posteromedial cornu of hypandrium; phallapodeme firmly fused to paired sclerotized bars, forming V- or Y-shaped “fultella” (term of Griffiths 1972), flexibly joined to the paired bar-like sclerites called “lateral sclerites of hypandrium” (McAlpine 1973) posteriorly fused to hypandrium and laterally connected to hypandrium by membrane; they are sometimes considered to be walls of modified, rudimentary gonites (Korneyev 1987) or gonocoxites (Korneyev 1999); in *Rivellia*, the left sclerite is completely fused with hypandrium (Korneyev 1985, Sueyoshi 2006), whereas in genera related to *Platystoma*, and in a few examined Scholastinae and Plastotephritinae both sclerites are symmetrical and free anteriorly (Korneyev 1985, 2001, Whittington 2003) membranous bottom of hypandrium allied to basiphallus in most “Higher Tephritoidea,” including Platystomatidae usually with a pair of small rounded sclerites (bearing fields of 8 trichoid mechanoreceptive sensilla), sometimes considered to be rudiments of parameres (Korneyev 1987) or gonopodites (Korneyev 1999). Epandrium dorsally setose, with lateral (or outer) surstyli fused to it without a seam, and connected either anteroventrally (Scholastinae, Plastotephritinae, *Rivellia* and related genera – see McAlpine 1973, Korneyev 1985, Hara 1989, Whittington 2003, Sueyoshi 2006, Galinskaya and Shatalkin 2013) or posteroventrally (*Platystoma* and related genera – see Hara 1987, Korneyev 2001). Ventrally of epandrium, a V-, X-, or H-like sclerite, the subepandrial sclerite (Cumming et al. 1995), sometimes referred as 10^th^ sternite or decasternum (e.g., Korneyev 1987) or, in its medial part, as “plate-like”, “transverse” or “bacilliform sclerite” (Sueyoshi 2006) is located between the hypandrium and cerci; Ovtshinnikova (1989, 1994) considered this sclerite to be a possible derivative of 10^th^ sternum and Cumming et al. (1995) considered this sclerite to be a possible derivative of sclerotized intersegmental membrane of the 10^th^ sternum. Its posterolateral lobes often form a pair of finger-like projections, called medial (inner) surstyli, bearing setae, including pair or subapical, dentate, thickened prensisetae; in Scholastinae and Plastotephritinae inner surstylus usually short, with prensisetae basal, whereas in most Platystomatinae, including *Platystoma*, *Rivellia* and related genera, the prenisetae are subapical, closer to apex of lateral surstylus (McAlpine 1973, Hara 1989, Korneyev 2001). Phallus consists of a basal ring-like, sclerotized basiphallus, flexibly joined to paired posterior arms of the phallapodeme, and a long tubular and coiled distiphallus, apically bearing a clearly expressed glans, well separated by a fold. Cerci usually large and apically widened (see McAlpine 1973, Korneyev 2001, Galinskaya and Shatalkin 2013)^1^.

### Musculature of the male genitalia

#### Rivellia alini

Taxon classificationAnimaliaDipteraPlatystomatidae

Enderlein, 1937

##### Material.

3 males: Russia, Primorsky Krai, Ussuri District, Kamenushka, 4 August 2013 (T.V. Galinskaya).

##### Description.

**Figure 1.**
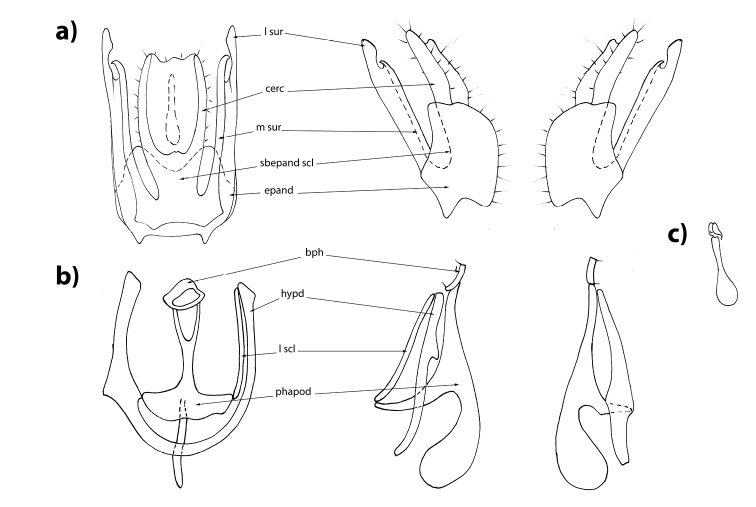
Male genitalia of *Rivellia alini* Enderlein, 1937. **a** epandrium in ventral, right lateral and left lateral views **b** hypandrium in dorsal, right lateral and left lateral views **c** ejaculatory apodeme.

Lateral sclerite of hypandrium separated from hypandrium at right side; left sclerite completely fused with hypandrium. Subepandrial sclerite consisting of the elongate bifurcated medial part and elongate postero-lateral lobes. Lateral surstylus long, apically curved. Cerci paired, long, sclerotized, ventrally connected to subepandrial sclerite (Figure 1).

**Figure 2.**
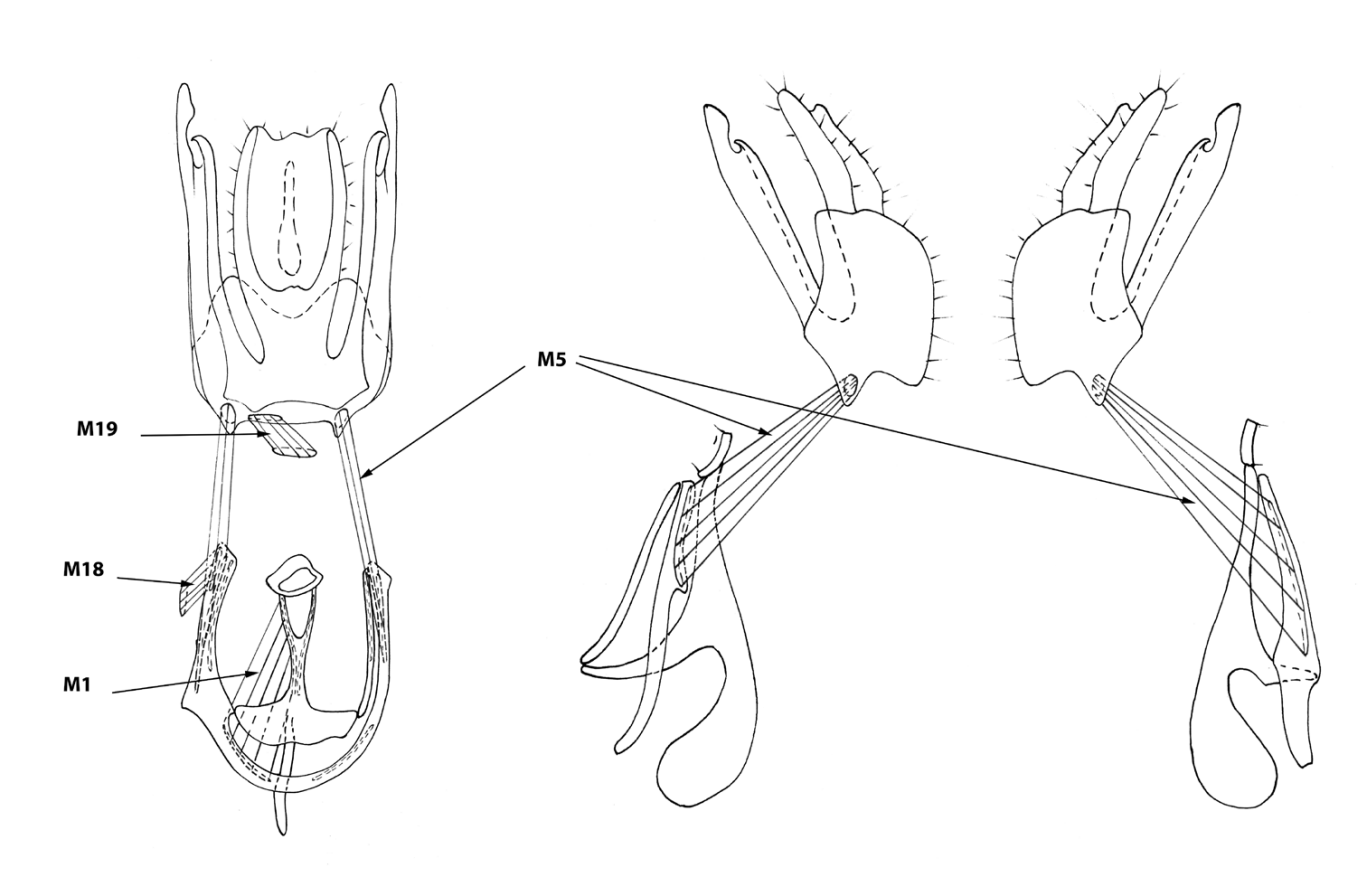
Male genitalia of *Rivellia alini* Enderlein, 1937. Epandrium and hypandrium in inner, right lateral and left lateral views.

**Figure 3.**
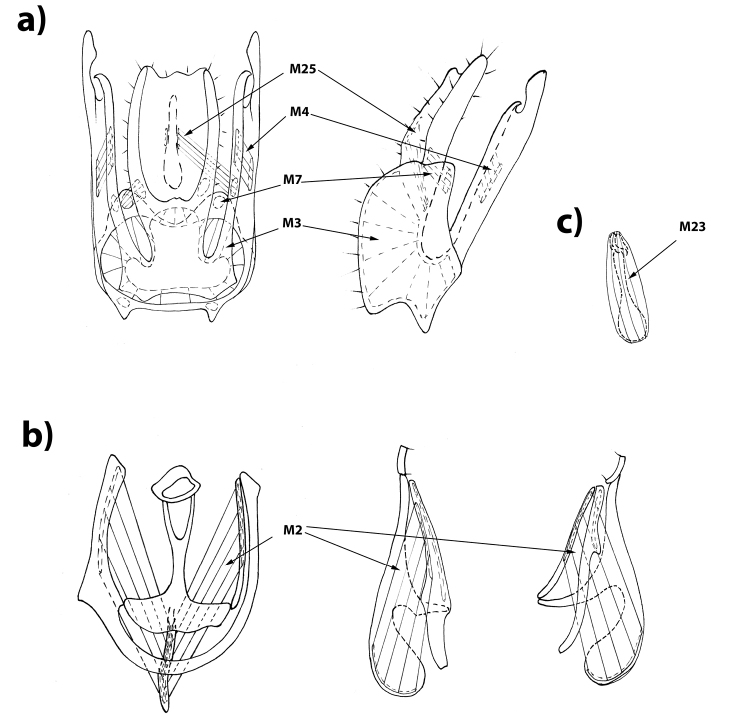
Male genitalia of *Rivellia alini* Enderlein, 1937. **a** epandrium in ventral and left lateral views **b** hypandrium in dorsal, left lateral and right lateral views **c** ejaculatory apodeme.

Platystomatidae have same set of muscles as in Ulidiidae, differing from them by the degree of development, shape, and their attachment sites (Figures 2, 3).

Muscles of the hypandrial complex: *M1*, *M2*, and *M23*. Strong, wide, and short phallapodeme retractors *M1* connect the anterior part of hypandrium with grooves on the Y-shaped phallapodeme arms. Strong, wide, and long phallapodeme protractors *M2* are attached to the distal half of the lateral surfaces of the unpaired phallapodeme lobe and to the inner surface of hypandrium at left and to the lateral sclerite at right side.

Ejaculator compressors *M23* strong and long, surrounding ejaculator apodeme. Their contraction pumps semen into the phallus.

Tergosternal muscles. Tergosternal muscles *M5* long, fan-shaped, connecting lateral parts of the epandrium anterior margin with lateral parts of anterior margin of hypandrium (in contrast to Ulidiidae, in which these muscles are attached to the distal margin of the hypandrium). These muscles draw hypandrium to epandrium. During copulation, epandrium clasps female’s ovipositor while the hypandrium is retracted into the male’s abdomen by contraction of these muscles.

Muscles of the epandrial complex: *M3*, *M4*, *M7*, and *M25*.

Subepandrial sclerite adductors *M3* strong, connecting the inner surface of the epandrium (occupying a considerable part of it) to the inner surface of anterior part of the subepandrial sclerite as a wide bundle.

Adductors of surstyli *M4* short, fine, poorly visible, extending from the middle of long posterolateral lobe of the subepandrial sclerite (medial surstyli) to the middle of lateral part of at inner surface of the (lateral) surstylus.

Cercus retractors *M7* short, fine, extending from the inner surface of the basal part of the epandrium to the basal cercal lobes.

The long and fine poorly visible retractors of anal integument *M25* connect the median part of medial surstyli with the basal part of the rectum.

Pregenital muscles: *M18^2^* and *M19*. The unpaired adductor of the hypandrium *M18^2^* extends from the distal part of syntergosternite 8 to the left hypandrial arm. The strong fan-shaped unpaired epandrial retractor *M19* obliquely extends from the distal part of syntergosternite 8 to the left part of the basal margin of the epandrium.

## Discussion and Conclusions

Our results were compared with musculature of male genitalia of *Rivellia basilaris* (Wiedemann, 1830) studied by Sueyoshi (2006). In this species he studied muscles of epandrial complex and tergosternal muscles and revealed three pair of muscles: *M42+43* (=*M4* sensu Ovtshinnikova), *M31* (=*M3*), *M34* (=*M5*). We confirmed his results and expanded the area of the study.

Comparisons of descriptions revealed homologies and the following correspondence between numbers of homologous muscles (Galinskaya and Ovtshinnikova 2014) (Table 2).

**Table 2.** Homologous muscles of Tephritoidea from different articles.

Number of muscle in current research	Numbers of homologous muscles	Muscle complex
*M1*	*MUS1* (Hanna 1938) and *M41* (Sueyoshi 2006)	Hypandrial complex
*M2*	*MUS2* (Hanna 1938) and *M35*–*37* (Sueyoshi 2006)	Hypandrial complex
*M23*	*MUS* (Hanna 1938)	Hypandrial complex
*M5*	*M34* (Sueyoshi 2006)	Tergosternal complex
*M3*	*M31* (Sueyoshi 2006)	Epandrial complex
*M4*	*M42+43* and *M44* (Sueyoshi 2006)	Epandrial complex
*M7*	*MC* (Sueyoshi 2006)	Epandrial complex

Analysis of the attachment sites of muscles has shown that in all studied families paired phallapodeme muscles *M2* are attached with one end to the distal half of the lateral surface of the unpaired lobe of phallapodeme, and with the other end it is attached either to the the inner surface of the hypandrial arms (in Ulidiidae; only on the left side, in *Rivellia*); they are attached to the lateral sclerites in some Tephritidae and (only on the right side) in *Rivellia*. Thus, the attachment of *M2* muscles to the lateral sclerites confirms their hypandrial origin.

It can be noted that the attachment sites of the subepandrial sclerite adductors *M3* and surstyli adductors *M4* are constant and these muscles are thus clearly distinguished from each other. These muscles are synergistic, and when they contract during copulation, the surstyli grasp and hold the female ovipositor, as they do in most other cyclorrhaphan flies, including those considerably different in the structure of the surstyli and subepandrial sclerite.

Comparative analysis shows that studied *Rivellia* displays similar sets of muscles of the male genitalia, close to the plan of structure fundamental for Cyclorrhapha, possibly as a result of reduction of or lacking some of the muscles (Ovtshinnikova 1989), and differs from this fundamental plan in the split of muscle *M3–4* into two pairs and presence of muscles of the anal integument *M25*, which is also typical of the family Syrphidae. We have also noted asymmetry in the muscles of genitalia.

In this paper we confirmed the hypandrial origin of lateral sclerite and or medial surstylus is a lobe derived from the bacilliform sclerite.

## Supplementary Material

XML Treatment for Rivellia alini

## Footnotes

1 The short characteristic of genital structures was kindly compiled by Valery A. Korneyev.
